# Bioenergetics and Gene Silencing Approaches for Unraveling Nucleotide Recognition by the Human EIF2C2/Ago2 PAZ Domain

**DOI:** 10.1371/journal.pone.0094538

**Published:** 2014-05-02

**Authors:** Mahmoud Kandeel, Abdullah Al-Taher, Remi Nakashima, Tomoya Sakaguchi, Ali Kandeel, Yuki Nagaya, Yoshiaki Kitamura, Yukio Kitade

**Affiliations:** 1 United Graduate School of Drug Discovery and Medical Information Sciences, Gifu University, Gifu, Japan; 2 Department of Biomolecular Science, Faculty of Engineering, Gifu University, Gifu, Japan; 3 Department of Physiology, Biochemistry and Pharmacology, Faculty of Veterinary Medicine and Animal Resources, King Faisal University, Alhofuf, Alahsa, Saudi Arabia; 4 Department of Pharmacology, Faculty of Veterinary Medicine, Kafrelshikh University, Kafrelshikh, Egypt; 5 Department of Biology, Faculty of Sciences and Arts, Alkamil Branch, King Abdul Aziz University, Alkamil, Saudi Arabia; 6 Faculty of Veterinary Medicine, Zagazig University, Zagazig, Egypt; Institute of Enzymology of the Hungarian Academy of Science, Hungary

## Abstract

Gene silencing and RNA interference are major cellular processes that control gene expression via the cleavage of target mRNA. Eukaryotic translation initiation factor 2C2 (EIF2C2, Argonaute protein 2, Ago2) is considered to be the major player of RNAi as it is the core component of RISC complexes. While a considerable amount of research has focused on RNA interference and its associated mechanisms, the nature and mechanisms of nucleotide recognition by the PAZ domain of EIF2C2/Ago2 have not yet been characterized. Here, we demonstrate that the EIF2C2/Ago2 PAZ domain has an inherent lack of binding to adenine nucleotides, a feature that highlights the poor binding of 3′-adenylated RNAs with the PAZ domain as well as the selective high trimming of the 3′-ends of miRNA containing adenine nucleotides. We further show that the PAZ domain selectively binds all ribonucleotides (except adenosine), whereas it poorly recognizes deoxyribonucleotides. In this context, the modification of dTMP to its ribonucleotide analogue gave a drastic improvement of binding enthalpy and, hence, binding affinity. Additionally, higher *in vivo* gene silencing efficacy was correlated with the stronger PAZ domain binders. These findings provide new insights into the nature of the interactions of the EIF2C2/Ago2 PAZ domain.

## Introduction

Gene silencing and RNA interference (RNAi) are cellular processes that control gene expression via the cleavage of target mRNA by either a cellular or synthesized small RNA [1], [2]. A small interfering RNA (siRNA) is loaded into an RNA-induced silencing complex (RISC) that guides the RISC complex to bind with the complementary mRNA sequence followed by endonucleolytic cleavage of the target mRNA, thus preventing gene expression [3]–[5]. RNAi technology has been found to be a useful strategy in the fight against cancers [6]–[8], viral infections [9]–[12], asthma [13], [14], rheumatoid arthritis [15], and other diseases [16], [17]. Eukaryotic translation initiation factor 2C2 (EIF2C2, Argonaute protein 2, Ago2) is an Argonaute (Ago) protein and considered to be the major player of RNAi.

Human Ago proteins are classified into the Ago and PIWI subfamilies. The Ago subfamily consists of four closely related proteins, namely, Ago1 (EIF2C1), Ago2 (EIF2C2), Ago3 (EIF2C3), and Ago4 (EIF2C4), while the PIWI subfamily includes HIWI, HILI, PIWIL3, and PIWIL4 [18]. Ago proteins have a conserved structure that is composed of two binding domains (PAZ and MID) and one catalytic (PIWI) domain [19]. The PIWI domain contains an RNase H fold and is considered as the catalytic RNA cleavage center of Ago proteins. The 5′-end of siRNA is anchored in the cavity of the MID domain, while the PAZ domain binds to the 3′-double nucleotide overhang of siRNA [20]–[24]. Interestingly, the function of the PAZ domain extends beyond a simple binding event to include significant effects on RNA loading and the slicer (endonuclease) activity of the RISC complex. PAZ-disrupted mutants of Ago proteins are unable to unwind or eject the passenger strand and prevent the formation of active RISC complexes [25]. Furthermore, chemical modifications of nucleotides that bind with the PAZ domain affect the overall RNAi process [26]–[28]. Within the Ago proteins of humans, EIF2C2/Ago2 is the only member with recognized slicer activity and is considered as the catalytic engine of the RNAi process. Furthermore, cells lacking EIF2C2/Ago2 are unable to process siRNA [29].

The characteristics and binding parameters of the PAZ domain (named after the three proteins that were initially found to contain this domain, such as PIWI, Ago, and Zwille) from Ago proteins have been characterized in *Drosophila melanogaster* Ago1 and Ago2 [22]–[24], [30], human EIF2C1/Ago1 [21], human HIWI [31], HILI, PIWIL3, and PIWIL4 [32], and mouse PIWIL1 [33]. However, the exact single nucleotide specificities of the PAZ domain are not well understood. The interaction of the 3′-end of RNA with the PAZ domain shows interesting molecular dynamics. During RNAi, the 3′-end of RNA toggles between binding and release from the PAZ domain in a cyclic manner. This process was demonstrated to be essential for proper RNAi and cleavage of the target RNA. In this context, we recently evaluated the impact of the strength of binding with the PAZ domain on the overall RNAi process using a computational approach [34], [35]. A small interaction surface and lower free binding energy were among the markers for favorable RNAi. To this end, there are still unknown factors in the recognition of RNA by the PAZ domain and several aspects of the modified 3′-end of siRNA have yet to be determined. Of special interest, the nature of nucleotides at the 3′-dangling end of siRNA is not well understood. In this study, we asked several questions to evaluate the exact molecular aspects of the PAZ domain. First, which of the known nucleosides can bind with the PAZ domain? Second, what are the determinants of nucleotide recognition by the PAZ domain? Lastly, what is the relationship between strong and weak binders and their *in vivo* RNAi efficacy? Here, we demonstrate that the EIF2C2/Ago2 PAZ domain has a selective binding affinity for ribonucleotides over deoxyribonucleotides. Furthermore, there was an inherent bias against adenine nucleotides. Within all ribonucleotides (rA, rG, rC, and rU), unexpectedly, rA showed very low affinity compared with the other ribonucleotides, which constitute the highest affinity ligands. We further show that the EIF2C2/Ago2 PAZ domain functions as an induced fit cavity and adapts to different nucleotides with no or minimal changes in conformation. Finally, we demonstrate that the higher binding nucleotides were generally associated with higher *in vivo* RNAi activity.

## Materials and Methods

### Design and synthesis of siRNAs

The used siRNAs were designed to suppress expression of *Renilla* luciferase. The 3′ overhang contained a single base extension of natural ribonucleotides or deoxynucleotides (Table 1). The **siRNAs 1–8** (**oligos no. 1–32**) were purchased from Hokkaido system science co. (Hokkaido, Japan). **Oligos no. 17** and **18** were synthesized with DNA/RNA synthesizer by using phosphoramidite method. Deprotection of bases and phosphates was carried out night in NH4OH/EtOH (3∶1) solution. 2′-TBDMS groups were removed by 1 M tetrabutylammonium fluoride in THF for 12 hours. The reaction was stopped by 0.1 M TEAA buffer pH 7 and desalted on Sep-Pack C18 cartiridge. Deprotected oligos were purified by 20% PAGE containing 7 M urea. The concentration of oligos was measured as OD units at 260 nm. The extension coefficient of the oligos was calculated from the mononucleotides and dinucleotides according to the nearest-neighbor approximation method. MALDI-TOF/MS analysis of oligos was performed to detect the mass of oligos with a time-of-flight mass spectrometer. siRNA9 was used in routine gel shift assays during purification of PAZ domain.

**Table 1:** Sequence of oligonucleotides used in this study.

No. of siRNA	Oligo. No.	Oligo	Sequence	No. of ON
siRNA1	ON 1	Renilla-rG AS	5′-GUAGGAGUAGUGAAAGGCCG-3′	20
	ON2	Renilla-rG S	5′-GGCCUUUCACUACUCCUACG-3′	20
siRNA2	ON3	Renilla-rU AS	5′-GUAGGAGUAGUGAAAGGCCU-3′	20
	ON4	Renilla-rU S	5′-GGCCUUUCACUACUCCUACU-3′	20
siRNA3	ON5	Renilla-rC AS	5′-GUAGGAGUAGUGAAAGGCCC-3′	20
	ON6	Renilla-rC S	5′-GGCCUUUCACUACUCCUACC-3′	20
siRNA4	ON7	Renilla-rA AS	5′-GUAGGAGUAGUGAAAGGCCA-3′	20
	ON8	Renilla-rA S	5′-GGCCUUUCACUACUCCUACA-3′	20
siRNA5	ON9	Renilla-dG AS	5′-GUAGGAGUAGUGAAAGGCCdG-3′	20
	ON10	Renilla-dG S	5′-GGCCUUUCACUACUCCUACdG-3′	20
siRNA6	ON11	Renilla-dT AS	5′-GUAGGAGUAGUGAAAGGCCT-3′	20
	ON12	Renilla-dT S	5′-GGCCUUUCACUACUCCUACT-3′	20
siRNA7	ON13	Renilla-dC AS	5′-GUAGGAGUAGUGAAAGGCCdC-3′	20
	ON14	Renilla-dC S	5′-GGCCUUUCACUACUCCUACdC-3′	20
siRNA8	ON15	Renilla-dA AS	5′-GUAGGAGUAGUGAAAGGCCdA-3′	20
	ON16	Renilla-dA S	5′-GGCCUUUCACUACUCCUACdA-3′	20
siRNA9	ON17	Renilla-F-dT AS	5′-F-GUAGGAGUAGUGAAAGGCCT-3′	20
	ON18	Renilla-F-dT S	5′F-GGCCUUUCACUACUCCUACT-3′	20

### Cell cultures and transfection

HeLa cells were grown at 37°C under optimal humidity and 5% CO_2_ in Dulbecco's Modified Eagle's Medium (D-MEM) supplemented with 10% bovine serum (BS). Twenty four hours before transfection, cells were diluted to 40000 cells/ml and aliquots of cells culture were transferred into 96-well plate (100 µl/well). Cells in each well were transfected with 35 µl of 20 ng psiCHECK-2 vector, 1 nM or 10 nM of each siRNA (siRNAs1–16), and 1.2 µg of transfection reagent in Opti-MEM Reduced Serum Medium. Cells were incubated for 24 hours and subjected to analysis of gene silencing efficacy. Control wells were adopted by transfection of cells in a mediun lacking siRNAs. After 1 hour, 100 µl MEM containing 10% BS was added to each well and incubated for 24 hours.

### Measurement of gene silencing

The ability of siRNAs to prevent gene expression was determined by dual-luciferase assay using psiCHECK-2 vector, which contains *Renilla* and firefly luciferase gene. The used siRNAs are targeting *Renilla* luciferase gene. Cell extracts were prepared in passive lysis buffer. The activities of *Renilla* and firefly luciferases in cell lysates were determined by a commercial Dual-Glo luciferase reporter assay kit. The signal of *Renilla* luciferase was compared with that of firefly luciferase from three different transfection experiments each contain three cultures for every oligo.

### Cloning, expression and purification of Ago2 PAZ domain

The Ago2PAZ domain was cloned into the BamHI and HindIII sites of pQE30 vector by using the standard cloning protocol. To generate PAZ domain, a fragment of Ago2 (residues 226–379) was amplified by using a pair of primers—forward (5′- ATAGGATCCGCACAGCCAGTAATCGAGTTTGTTTG-3′) and reverse (5′- GCGAAGCTTAATCTCTTCTTGCCGATCGGGC-3′). The recombinant *E. coli* cells were grown in LB medium containing 50 µg/ml ampicillin at 37°C to the mid-log phase (*D*_600_ = 0.6). Induction of expression was carried out by the addition of IPTG (isopropyl β-D-1-thiogalactopyranoside) to a final concentration of 0.5 mM and cell growth was continued at 20°C for 8 h. Cells were lysed in an extraction buffer (25 mM potassium phosphate buffer pH 7). After extraction, ethyleneimine polymer was added to a final concentration of 0.05% to precipitate nucleic acids. The hexahistidine-tagged PAZ domain was purified from the soluble cell extract by using talon metal affinity resin followed by gel filtration chromatography by using sephacryl S200HR. A final polishing step was performed by using Amicon Ultrafree-MC centrifugal filter unit (5 kDa membrane cut off value).

### Isothermal titration microcalorimetry

Calorimetric experiments were carried out using VP-ITC. Before ITC experiments, PAZ domain was thoroughly dialyzed against ITC buffer composed of 25 mM potassium phosphate buffer pH 7. After dialysis, PAZ solution was centrifuged at 15000 RPM for 20 min and the precipitate was discarded. Concentrated PAZ solution was kept at 4°C and diluted with the final dialysis solution immediately before use in ITC titrations to a final concentration of 30 µM. Substrate solution was made from the final dialysis buffer of proteins to minimize artifacts due to subtle differences in buffer composition. The protein solution was thoroughly degased and loaded into the sample cell. The substrate solution was loaded into the syringe and used to titrate the protein solution by twenty sequential injections. Control experiments were performed by injecting the nucleotide into the dialysis buffer to determine the heats of dilution. The apparent heat change after each injection was integrated and corrected for the heat of dilution of the nucleotide. The data was fitted to a single-binding site model by non-linear regression analysis to yield the thermodynamic parameters *K_a_*, association constant; Δ*H*, enthalpy of binding; and n, the stoichiometry of binding. The affinity of the nucleotides to protein is given as the dissociation constant (*K_d_* = 1/*K_a_*). The binding entropy contributions were calculated from the equation Δ*G* = Δ*H*–*T*Δ*S* and Δ*G = -RT* ln *K*, where *K* is the association constant, *T* is the absolute temperature, Δ*S* is the entropy change, and Δ*G* is Gibb's free energy change.

### Heat capacity measurement

To address the association between structural and thermodynamic data, ITC titration experiments were conducted at four different temperatures between 8 and 30°C and the thermodynamic parameters were determined. The change in heat capacity was determined from the temperature dependence of the enthalpy according to the following equation: Δ*Cp* = δΔ*H*/δΔ*T* (1)

### Mutual competitive displacement experiments

Free PAZ domain was titrated with AMP, dAMP, dCMP, uridine or thymidine until reaching saturation. After finishing the first experiment, the ITC syringe was removed, thoroughly washed and loaded with UMP and a second titration experiment was carried out. All competitive binding experiments were carried out at 12°C. For simulation of competitive binding data, analysis model was used as previously described.

### Structure-thermodynamic calculations

To test whether the binding of nucleotides with PAZ domain is associated with changes in the proteins accessible surface area, we analyzed several crystal structures by using VADAR web server (Volume, Area, Dihedral Angle Reporter). The Δ*C_p_* was calculated using the following equation: Δ*C_p_* = 0.45 ΔΔASAapolar–0.26 ΔΔASApolar (2), where ΔΔASAapolar and ΔΔASApolar are the changes in ASA of polar and apolar residues, respectively.

From the output of data, we selected and compared the total and fractional values of surface polar and nonpolar surface areas. The obtained data were used in empirical equations to analyze the change in surface area and its components after binding with the substrates.

### Synthesis of 5-methyl uridine monophosphate (rTMP)

During the ITC assay, the binding affinity of deoxythymidine nucleoside was surprisingly low. Furthermore, dTdT is a standard 3′-terminal additive in many RNAi assays. Binding affinity studies revealed that the PAZ domain bears an inherently high affinity for ribonucleosides (except rA). Therefore, we designed a new ITC experiment to check the hypothesis that the PAZ domain preferentially binds ribonucleosides (except rA), regardless of the attached base. Moreover, this modification will give us an insight into the functional role of the 2′-OH of nucleotides. In this context, 5-methyl uridine was considered as a thymidine base attached to a ribosugar. Thus, a new comparison was carried out between deoxythymidine and ribothymidine nucleosides.

5-Methyluridine-5′-O-monophosphate was prepared as follows. Distilled H_2_O (28 µL, 1.56 µmol), MeCN (2.38 mL), pyridine (215 µL), and POCl3 (206 µl, 2.21 mmol) were added to a 20-mL round-bottom flask on ice, then 5-methyluridine (1) (100 mg, 387 µmol) was added. The mixture was stirred at 0°C under argon gas for 5 h. The reaction was moved to room temperature and stirred for an additional 43 h. The reaction was checked by TLC (BuOH: H2O: acetic acid  = 2∶2∶1), and quenched by iced H2O (1 mL). The mixture was concentrated and the residue was purified by ion exchange column chromatography (0–5% formic acid in H2O), then the eluate was neutralized by NH4OH (1 mL). The desalted supernatant solution was concentrated to give 5-methyluridine-5′-O-monophosphate (2) (98.4 mg, 291 µmol, 75%) as a brown solid. The resulting compound was checked by NMR (Figure S1).

### Statistical analysis

The statistical analysis of RNAi potency was carried out using STATA version 12.1. A single-tailed one-way analysis of variance with Bonferroni's multiple comparison test was conducted. The results of statistical analysis are provided in Tables S1 and S2.

### Other methods

Protein concentration was determined using a protein assay reagent with bovine serum albumin as the standard. The molecular mass of the recombinant enzyme was determined by SDS-PAGE (15% polyacrylamide). After electrophoresis, the gel was stained with Coomassie Brilliant Blue R-250. For Western blotting, purified protein was applied to 15% SDS–PAGE, transferred to a PVDF membrane, and detected by anti-His tag (10,000-fold dilution). The immunoprobed proteins were detected using an HRP conjugated substrate detection kit.

## Results

### Isothermal titration calorimetry

**Figure 1:**
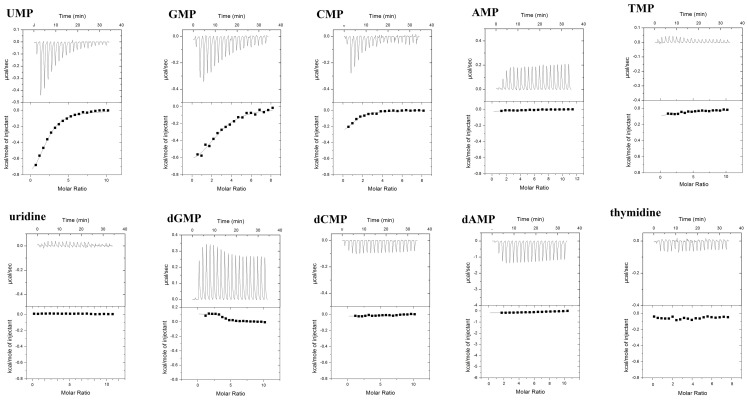
Representative ITC profiles of the binding of nucleotides to the free PAZ domain. The top panels show the raw heat change data. The lower panel shows the integrated binding isotherms as a function of the molar ratio of the ligand to the enzyme.

ITC has largely became the method of choice in determining the binding affinity of two interacting partners [36]–[39]. Furthermore, in one experiment we can get the full thermodynamic profile associated with binding. In this work, ITC was carried out for determining the binding parameters as well as the mechanisms of interaction of different nucleosides with Ago2PAZ domain. The typical ITC titrations included addition of the protein in the cell and titrated with the ligand in a time controlled process. So that, if a binding event is likely to occur, the initial titrations will give high exothermic or endothermic peaks. As the titrations proceed, the binding sites will be gradually occupied by the ligand giving rise to smaller peaks. In the last injections, as the binding sites are completely occupied, the heat arising from injections are similar to the heat of background. Figure 1 shows the typical titration of Ago2PAZ domain with the nucleotides. The top panel shows the raw calorimetric data referring to the amount of heat produced following each injection. The bottom panel shows the integrated amount of heat generated per injection as a function of the molar ratio of ligand to protein. The obtained thermodynamic constants are given in Table 2.

**Table 2:** The thermodynamic constants obtained by isothermal titration calorimetry for the association of nucleosides with Ago2PAZ domain.

Substrate	*n*	*K_a_*	*K_d_*	Δ*H*	Δ*G*	*T*Δ*S*	Δ*S*
		M^−1^*10^4^	(µM)	(kcal/mol)	(kcal/mol)	(kcal/mol)	(cal/K mol)
UMP	2.2±0.5	6.6±0.6	15	−0.9±0.1	−6.4	5.3±0.3	18.2
GMP	2.7±0.5	4.5±0.3	22	-0.78±0.05	6.1	5.3±0.3	18.5
CMP	2.7±0.5	2.5±0.2	40	−0.37±0.02	−5.8	5.4±0.4	18.8
TMP	1.7±0.4	0.4±0.3	232	0.52±0.02	−4.8	5.3±0.3	18.5
dGMP	2±0.4	2.5±0.1	40	0.1±0.001	−5.8	5.9±0.4	26.6
AMP/dAMP/dCMP	ND	ND	ND	ND	ND	ND	ND
Uridine/thymidine	ND	ND	ND	ND	ND	ND	ND

The one set of sites binding model is used for fitting the data. (n = 3).

The isothermal titration calorimetry (ITC) assays included the ribonucleoside monophosphates UMP, GMP, CMP, and AMP, the deoxynucleosides monophosphates dTMP, dGMP, dCMP, and dAMP, and nucleosides such as uridine and thymidine. No binding activity was measured with thymidine or uridine (Fig. 1, Table 2), indicating the importance of nucleoside derivatives; for example, phosphate, in the recognition of substrates by the PAZ domain. Within ribonucleotides, binding activity was detectable with UMP, GMP, and CMP. Surprisingly, AMP binding was not measurable, indicating no or very weak binding of AMP with the PAZ domain. Within the deoxyribonucleotides, dGMP showed the most marked binding affinity. Furthermore, the nucleoside thymidine, which is used as a standard nucleotide at the 3′-overhang of siRNA in many RNAi experiments, showed very low binding activity.

#### Thermodynamic signature

The thermodynamic signatures of UMP, GMP, CMP, TMP, and dGMP binding with the EIF2C2/Ago2 PAZ domain indicated different binding patterns among the ribonucleosides and deoxyribonucleosides (Figure S2). The thermodynamic signature of nucleosides included a negative Δ*G*, positive TΔ*S*, and negative Δ*H* for UMP, GMP, and CMP, and a positive Δ*H* for dTMP and dGMP. A negative Δ*H* value indicates favorable enthalpic conditions, while a positive value indicates unfavorable enthalpic conditions. Furthermore, a positive TΔ*S* value indicates favorable entropic conditions. Favorable enthalpic and entropic terms are the most favorable thermodynamic profiles for tight binding. Therefore, the weak binding of dTMP and dGMP is favored by adverse enthalpic conditions due to their positive Δ*H* values.

#### Substrate binding affinity

UMP showed the highest binding affinity (with *K_d_* value of 15 µM) followed by GMP, CMP and dGMP, respectively (Figure S3, Table 2). TMP showed at least 15-fold increase in the dissociation constant compared with UMP.

#### Binding enthalpy and entropy

The interaction of ribonucleotides (except rA) was characterized by low negative enthalpy with Δ*H* value <1 kcal/mol (Table 2). In contrast, the deoxyribonucleosides dTMP and dGMP showed a low positive enthalpy change. The positive Δ*H* value indicated unfavourable enthalpic conditions.

### Mutual competitive binding experiments

**Figure 2:**
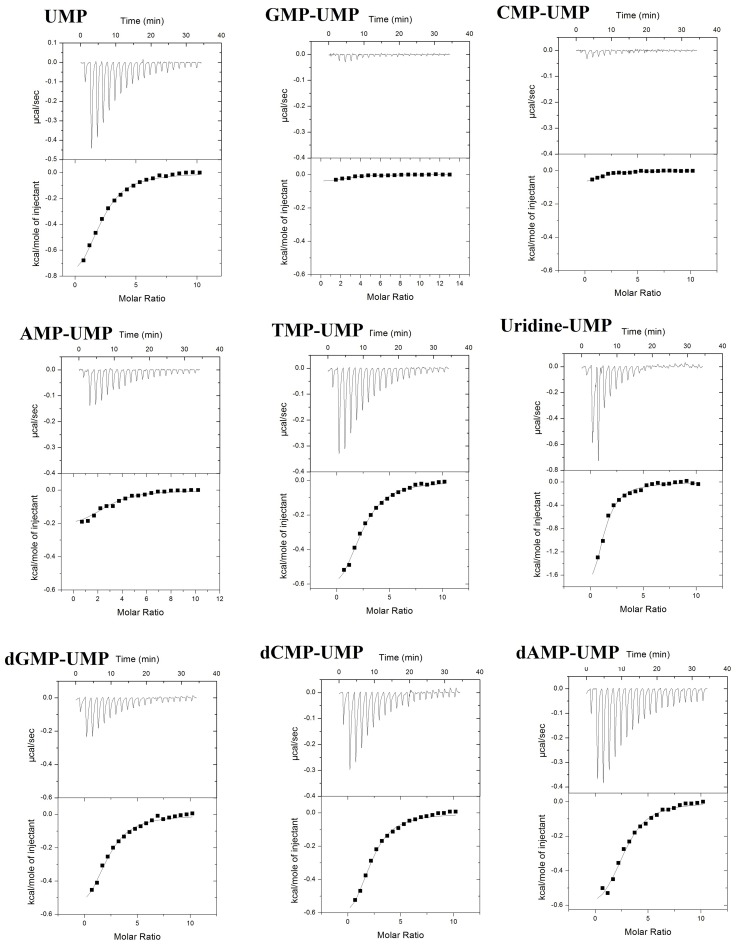
ITC profiles of the binding of nucleotides with the PAZ domain bound with various nucleotides.

In a mutual competitive binding experiment, we assumed that there was residual binding of the ribonucleotide AMP and the deoxyribonucleotides dAMP and dCMP or uridine nucleoside with the PAZ domain. Thus, two sequential experiments were performed. The first experiment included the titration of the first (low or no affinity) ligand followed by titration with UMP. If binding of AMP, dAMP, dCMP, or uridine is likely to occur, the binding of UMP could be minimized by the degree of binding of the other ligand. As a control, the same experiments were carried out in the presence of stronger binding ligands such as GMP. Figure 2 shows the results of the competitive binding experiments. Titration of the EIF2C2/Apo2 PAZ domain with UMP gave the characteristic figure shown as (UMP). Titration with UMP into a preformed complex of the PAZ domain with GMP (GMP-UMP) or CMP (CMP-UMP) showed very weak signal without measurable thermal parameters, corresponding to a lack of UMP binding. Furthermore, in the presence of the medium affinity binding ligand dGMP (dGMP-UMP) or TMP (TMP-UMP), UMP showed a corresponding decreased access to the binding site as evidenced by lower exothermic peaks compared with the control (UMP). Finally, there was a slight decrease in the affinity of UMP in the presence of other ligands (dCMP, dAMP, and uridine). The calculated values of the weak binding ligands are given in Table 3. The estimated values of such low affinity ligands were 72–159 M^−1^, while uridine was not expected to bind with the PAZ domain.

**Table 3:** The thermodynamic constants obtained by isothermal titration calorimetry for the association of nucleosides with Ago_2_PAZ domain.

Substrate	*n*	*K_a_*	*K_d_*	Δ*H*	Δ*G*	*T*Δ*S*	Estimated *K_B_*
		M^−1^*10^4^	(µM)	(kcal/mol)	(kcal/mol)	(kcal/mol)	M^−1^
UMP	2±0.1	6.6±0.6	15	−0.97±0.1	−6.4	5.3±0.3	-
AMP-UMP	2.7±0.2	4.9±0.5	20	−0.24±0.2	−6.5	6.3±0.3	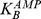 = 143
dAMP-UMP	2.7±0.2	6.2±0.5	62	−0.68±0.5	−6.6	6±0.3	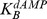 = 72
dCMP-UMP	2.1±0.1	5.6±0.4	17.8	−0.75±0.6	−6.6	5.8±0.2	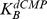 ** = **155
Uridine-UMP	1.2±0.02	9.9±0.8	10	−1.9±0.05	−6.9	5.1±0.1	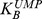 = ND

The one set of sites binding model is used for fitting the data. (n = 3).

### UMP/GMP-induced heat capacity change (Δ*C_p_*)

The measurement of heat capacity is an important parameter in determining the structural changes of the PAZ domain in response to its ligands. Δ*C_p_* can be determined by running the typical ITC experiment at different temperatures. For this purpose, the PAZ domain was titrated with UMP or GMP and the change in enthalpy was recorded at the different temperatures (Table 4).

**Table 4:** The thermodynamic constants obtained by isothermal titration calorimetry for the association of nucleosides with Ago2PAZ domain.

substrate	*T*	*K*a	*K*_d_	Δ*H*	Δ*S*
	°C	M^−1^*10^4^	(µM)	(cal/mol)	(cal/mol)
UMP	8	6.3±0.4	15.8±0.5	−1230±66	21±1
	15	1.6±0.2	62.5±1.5	−238±15	18.4±1.1
	30	1.3±0.1	77±2.2	−89±5	12±1.2
GMP	8	4.9±0.2	20±1	−251±20	20.6±1
	15	4±0.45	25±1.2	−173±10	21.4±1.1
	30	1.9±0.1	52±4	−84±0.5	12.5±0.8

The one set of sites binding model is used for fitting the data. (n = 3).

At lower temperatures, the binding of UMP or GMP was under enthalpic forces due to their negative Δ*H* values. Furthermore, there was a gradual decrease in enthalpic forces as the temperature increased. The enthalpy changes of UMP Δ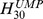/Δ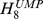 and Δ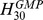/Δ
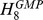
were 13.6 and 3, respectively. Thus, the enthalpy change decreased by approximately 13-fold when the temperature was raised from 8 to 30°C. The decrease in enthalpic change resulted in a small positive value of Δ*C_p_* (equation 1). The calculated Δ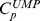 and Δ
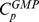
values were +51 and +7.5, respectively.

### Binding parameters of 5-methyl uridine monophosphate (rTMP)

To understand the biological significance of the presence of 2′-OH in TMP, we synthesized rTMP (Figure S1). Titration of the PAZ domain with rTMP produced a remarkable exothermic signal in comparison with dTMP (Figure S4). The binding affinity of rTMP was in the micromolar range with a *K_a_* value of 17000 M^−1^, indicating a 4-fold increase in binding affinity compared with dTMP. The interesting feature is the change of the enthalpic components of rTMP. While the binding of dTMP was enthalpically unfavorable (+520 cal/mol), rTMP binding showed favorable enthalpic conditions with a Δ*H* of −64 cal/mol.

### Gene silencing assay

**Figure 3:**
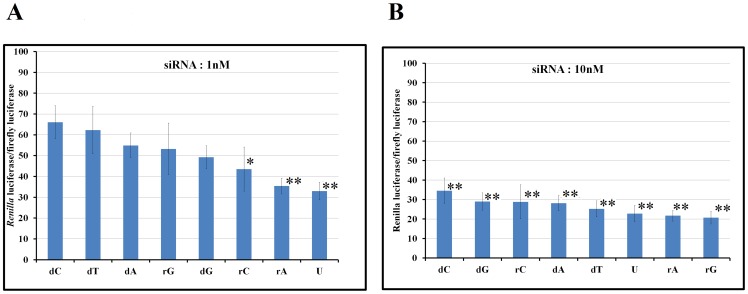
Dual luciferase assay. The materials and conditions are described in the Methods section. *Significant at 0.05 level. **Significant at 0.01 level.

The gene silencing capability of siRNAs was estimated using a dual-luciferase assay. siRNAs that contained ribonucleotides showed more potent RNAi activity than siRNAs that carried deoxynucleotides at their 3′-overhang (Fig. 3). Furthermore, the siRNA data bear an interesting correlation with data from ITC. The strongest RNAi was shown by rG or uridine, while low RNAi efficiency was produced by dC (Fig. 3). At 10 nM, all treatments showed a statistically significant RNAi effect compared with the control. Interestingly, at a 10-fold lower concentration (1 nM), only siRNAs bearing ribonucleotides showed statistically significant results compared with the control (Tables S1 and S2).

## Discussion

The PAZ domain is a universal binding pocket and adapts to a number of nucleotides with a wide range of binding affinities

The initial ITC experiments showed that some nucleotides (UMP, GMP, CMP, TMP, and dGMP) bound to the PAZ domain with considerable affinity. The lack of estimation of binding of some other ligands with the PAZ domain does not necessarily exclude the occurrence of a binding event. For instance, these substrates might bind with very low affinity (beyond the accurate detection window of ITC); furthermore, the substrate might be negatively adsorbed onto the surface of the PAZ domain without a detectable exothermic interaction. To distinguish between these possibilities, the ITC experiments were repeated in the mutual displacement mode. At first, the binding constant and the thermodynamic parameters of the binding of the high affinity ligand (UMP) with the free protein were estimated. Then, we repeated the same experiment by titrating UMP in the presence of the PAZ domain bound with the low affinity ligand to check whether the affinity of UMP was changed in the presence of the other ligand. The difference between these two titrations can be used to detect the binding parameters of the low affinity ligand according the following equations [40]: K_app_ = K_A_/1+K_B_[B] (3) and ΔH_app_ = ΔH_A_-ΔH_B_(K_A_/1+K_B_[B]) (4), where *K_A_* is the equilibrium constant for the tightly binding ligand, *K_B_* is the equilibrium constant for the weakly binding ligand, *K_app_* is the binding constant of the tightly binding ligand in the presence of the weakly binding ligand, Δ*H_B_* is the enthalpy change accompanying the weakly binding ligand and Δ*H_A_* is the enthalpy change accompanying the tightly binding ligand and [*B*] is the concentration of the free weakly binding ligand.

According to the ITC-measured dissociation constants, the nucleotides binding with the PAZ domain can be classified into three grades. Grade 1 ligands: medium affinity group includes UMP, GMP, CMP, and dGMP in which the affinity constant was >10000 M^−1^. Grade 2 ligands: include low-medium affinity ligands such as TMP. Grade 3 ligands: are the low affinity ligands and include AMP, dAMP, and dCMP.

### Mechanism of interaction of nucleotides with PAZ domain

To elucidate the possible forces contributing to the recognition of nucleotides, we analyzed and correlated the binding site data with the thermal data. Alignment of the sequences of PAZ domain from different Argonaute proteins is given in Figure S5. The cavity enclosing the nucleotides (amino-acids enclosed in green boxes) is almost composed of hydrophobic residues with a minor contribution from hydrophilic amino-acids. Furtheremore, the hydrophobic residues constitute the direct contact with nucleotides (F68, F86, H110, T111 and Y112). Interestingly, the high conservation of nucleotide-binding amino-acids in PAZ domains indicates a common PAZ domain binding pattern among different Agos.

### The base or sugar of nucleotides, which is critical for binding with the PAZ domain?

With respect to the size of the nucleotide, there was no discrimination based on their size. The small bases, such as cytidine, uridine, and thymidine in rCMP and UMP, were bound to the cavity of the PAZ domain with an affinity comparable to that produced by the larger purines, such as guanosine of GMP. Therefore, the size of the base attached to the nucleoside is not the critical factor for binding with the PAZ domain. On the other hand, we observe a different phenomenon when we compared the functional type of the sugar ring attached to the nucleic acid base. It was apparent that the PAZ domain has different binding preferences, such as being selective for ribonucleotides over deoxyribonucleotides. For instance, the affinities of dGMP and dCMP were far lower than those of their respective ribonucleotides.

**Figure 4:**
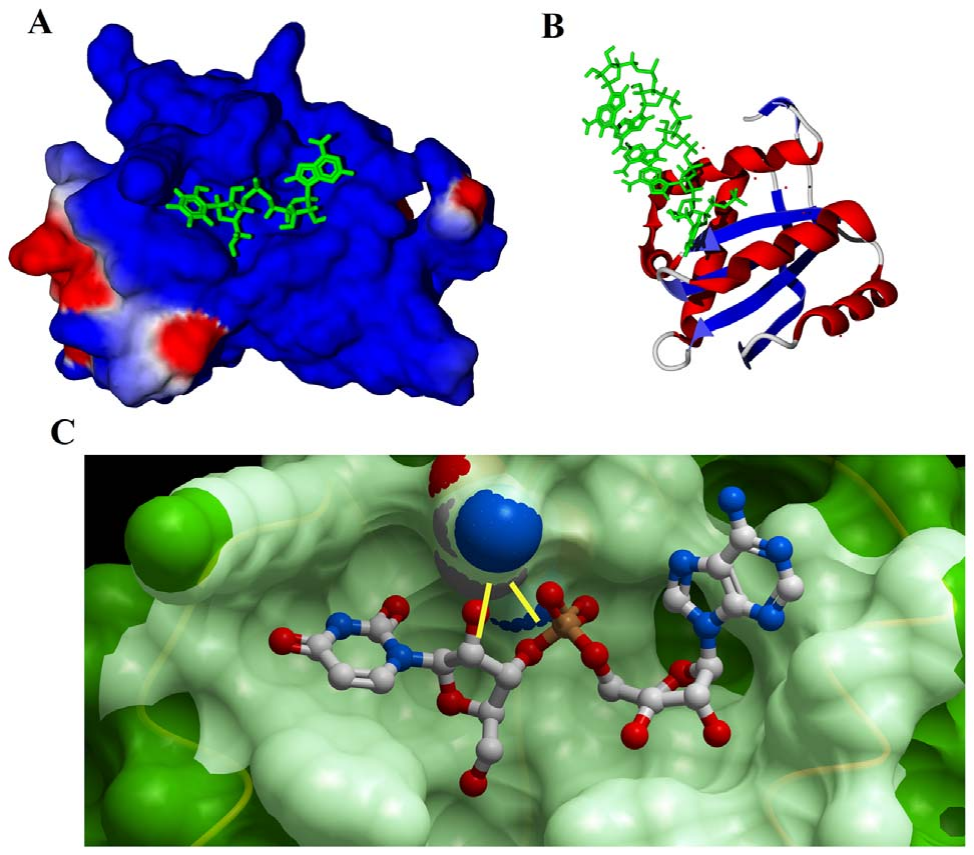
Surface representation of the interaction of nucleotides with PAZ domain of Drosophila. Accessible surface representation of the *Drosophila* Ago2 PAZ domain showing the binding cavity of the Ago2 PAZ domain bound with CMP at its 3′-terminal (A). Ribbon representation of the PAZ domain bound with siRNAis shown in (B). Hydrogen bonds (shown in yellow) between the ribose of CMP and the side chain of GLN548 (C). The coordinates of interaction were derived from PDB ID 4F3T.

**Figure 5:**
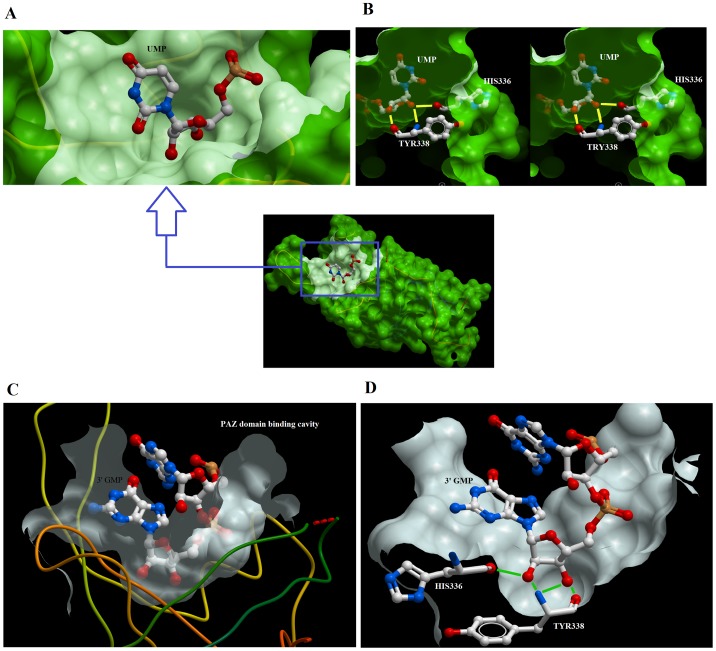
Surface representation of the interaction of nucleotides with PAZ domain of Human Ago2. Accessible surface representation of the human EIF2C2/Ago2 PAZ domain (A) showing the binding cavity of the PAZ domain bound with UMP at its 3′-terminal. Stereoview showing the hydrogen bonding of the ribose sugar with the side chains of HIS110 and TYR112 (B). The binding cavity of the EIF2C2/Ago2 PAZ domain bound with GMP at its 3′-terminal (C). Hydrogen bonds between the ribose of GMP and the side chains of HIS110 and TYR112 are shown in green (D). The coordinates of interaction were derived from PDB ID 4EI1 and 4F3T. The figure was generated using Molsoft.

From the thermodynamic experiments, it was apparent that the absence of 2′-OH adversely affected the binding of nucleotides with the PAZ domain, probably due to the loss of important hydrogen bonding with the PAZ domain. This was manifested by the unfavorable enthalpic conditions accompanying dGMP and dTMP binding. The crystal structure of human EIF2C2/Ago2 was resolved when either uridine or guanosine nucleotides were bound to the PAZ domain (PDB ID 4EI1 and 4F3T, respectively). Examination of the structure of the *Drosophila* Ago2 PAZ domain with oligonucleotides reveals that the first nucleotide and, to a lesser extent, the second one, interact with the PAZ domain (Fig. 4A), while the rest of the sequence faces the solvent (Fig. 4B). Within this configuration, the 2′-OH of the ribose ring interacts with glutamine (GLN548) through hydrogen bonding (Fig. 4C). Examination of the human EIF2C2/Ago2 PAZ domain structure bound with uridine nucleotide reveals that the 3′ UMP is engulfed within a deep cavity of the PAZ domain (Fig. 5A). UMP adopts a U-shaped structure with the sugar ring at the bottom and is swallowed by the cavity. Furthermore, both the 2′- and 3′-OH groups of the sugar ring are bound to the PAZ domain through bidentate hydrogen bonds with the side chains of HIS110 and TYR112 (Fig. 5B). A similar configuration was also found for the binding of guanosine nucleotides (Fig. 5C) in which bidentate hydrogen bonds were evident between the hydroxyl groups of the ribose and the side chains of HIS110 and TYR112. Such interactions of the 2′-OH of ribose provide the basis for RNA selection and lower affinity of the PAZ domain for deoxyribonucleotides.

In order to check our hypothesis that the nucleotide-bound sugar ring is the critical factor for EIF2C2/Ago2 PAZ domain binding regardless of the type of attached nucleic acid base, we synthesized rTMP and compared its binding parameters with dTMP. rTMP is an analogue of dTMP that contains a ribose sugar instead of the deoxyribose of dTMP. Thermodynamic data indicated drastic changes in binding affinity after the addition of 2′-OH of the synthesized rTMP. Furthermore, there were favorable enthalpic conditions that probably arose from the ability of rTMP to make hydrogen bonds with the side chains of HIS110 and TYR112. The large difference in the enthalpic components of dTMP and rTMP suggests that other favorable enthalpic forces are more important than those formed between 2′-OH and the side chains of HIS110 and TYR112. The presence of 2′-OH in ribonucleotides did not only facilitate binding through hydrogen bonds with the PAZ domain but also allowed a favorable conformation of the nucleotide for other binding sites, which are eventually lost with deoxyribonucleotides. Recently, the impact of 2′-OH modifications of siRNA on yeast Ago1 activity was investigated [41]. Interestingly, the substitution of 2′-OH with 2′-H at several positions, including the nucleotides binding to the PAZ domain, resulted in a significant impairment of yeast Ago1 activity. Together, these further clarify the role of 2′-OH not only for recognition by the PAZ domain but also for the overall activity of Ago proteins.

### Bias of the EIF2C2/Ago2 PAZ domain against adenine nucleotides

**Figure 6:**
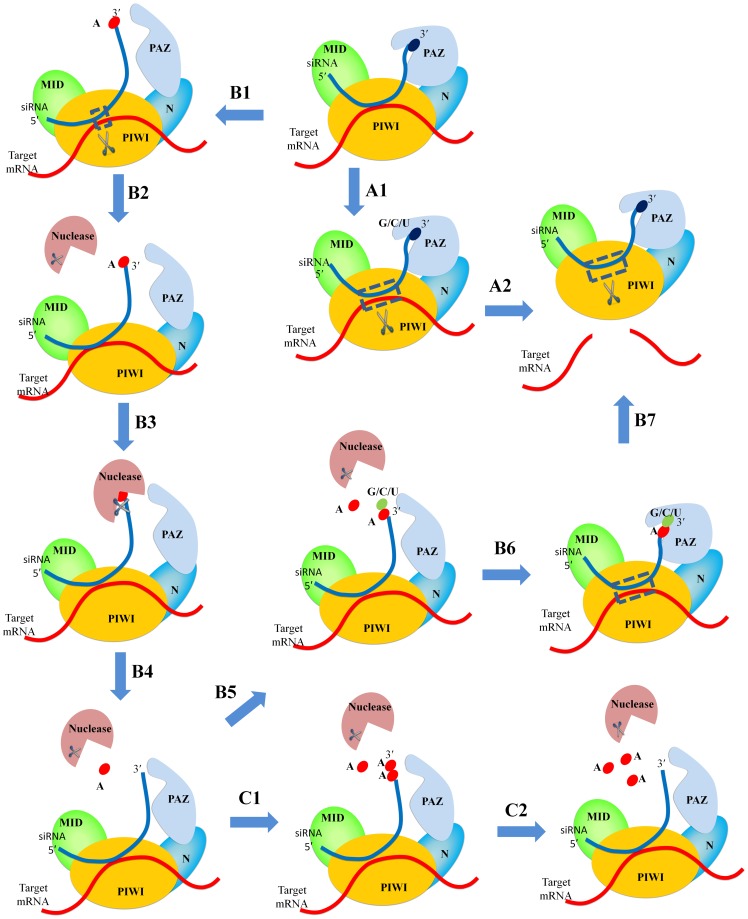
Model outlining the possible interactions of nucleotides at the 3′-terminal of siRNA.

The binding data revealed that all forms of adenine nucleotides showed the lowest binding efficiency among the tested nucleotides. The bias of cellular proteins against certain nucleotides was reported previously [42]. Specially referring to EIF2C2/Ago2, several reports indicated an unusual association between EIF2C2/Ago2 and adenine nucleotides. In one study, the trimming behavior of a set of miR-124 mutant constructs with the 4 different nucleotides at their 3′-terminal was investigated [43]. Cells overexpressing EIF2C1/Ago1, EIF2C3/Ago3, and EIF2C4/Ago4 did not show inherent cleavage affinity to any of the terminal nucleotides. In contrast, EIF2C2/Ago2-overexpressing cells showed high cleavage activity of the mutant miR-124 that contained adenosine nucleotides at its 3′-terminal. Furthermore, the cleaved product was one nucleotide shorter than the miR-124 mutants containing G or C or U at their 3′-terminal. Additionally, trimming of the 3′-terminal was in the order A<C<G<U. Consistent with the model provided by Juvvuna et al. [43], in which the highest exposure to trimming exonucleases in Ago2-loaded miRNA was associated with A nucleotides and the lowest rate was with U nucleotides, we assume that the very low affinity of A nucleotides allowed a weak interaction with the PAZ domain and they became vulnerable to exonucleases. In this regards, based on our thermodynamic data, we suggest a model for the cleavage of nucleotides at the 3′-terminal (Fig. 6). The presence of adenosine results in loose binding with the PAZ domain, making it vulnerable to cleavage by cellular nucleases. In contrast, the strong binding of U/G/C nucleotides does not allow the cleavage process to occur. In another study, the correlation between the adenylation and uridylation of RNAs with Ago-specific binding was investigated [44]. The authors reported that adenylation appeared to reduce the effectiveness of the miRNA targeting of mRNA transcripts. Furthermore, miRNA bound with EIF2C2/Ago2 had a low number of adenine nucleotides at its 3′-terminal relative to miRNA extracted from the whole cell. Therefore, on the basis of our data, we suggest that there may be an usual terminal adenylation rate occurring at the 3′-end of RNAs in the cell; however, the fraction bound with EIF2C2/Ago2 is the smallest among Ago proteins due to its inherent low affinity for adenine nucleotides.

Combining these previous two examples together, either trimming or extension of the 3′-terminal shared a common characteristic, that is the presence of an adenine nucleotide at this site infers a common fate. The presence of adenine nucleotides at the 3′-end of an miRNA improves its trimming or decreases its binding with the EIF2C2/Ago2 PAZ domain.

Reviewing the previous observations as well as our ITC data, which indicate the lowest binding affinity for adenine nucleotides, there could be an inherent bias for the EIF2C2/Ago2 PAZ domain against adenine nucleotides.

On the basis of the previously discussed data, we suggest a model outlining the possible interaction of nucleotides at the 3′-terminal of siRNA (Fig. 6). There are two possibilities for nucleotides at the 3′-terminal of siRNA; either strong binders such as G, C, or U nucleotides (Fig. 6 A1) or weak binders such as an A nucleotide (Fig. 6 B1). In the presence of strong binders, the PAZ domain and EIF2C2/Ago2 adopts a favorable conformation for proper RNAi (A1 and A2). In contrast, the A nucleotide (B2) binds loosely or not at all to the PAZ domain, thereby becoming vulnerable to cleavage by nucleases and thus exposing the second nucleotide in the siRNA sequence (B3, B4). The process of cleavage can be repeated if the following nucleotide in the sequence is also an A nucleotide (C1 and C2); otherwise, stronger binders can interact with the PAZ domain and induce RNAi (B5 and B6). To this end, we would expect different and interesting interactions of siRNAs with the PAZ domain according to the nature of the nucleotides present at the 3′-terminal of the siRNA.

### The EIF2C2/Ago2 PAZ domain is an induced-fit nonflexible binding cavity

The energetic parameters of substrate-receptor binding allows us to estimate whether the binding mechanism operates by an induced fit process or is accompanied by conformational changes. The characteristic behavior of the induced fit process comes from the presence of high entropic barriers. The changes in ΔS along the measured experimental temperatures were very small, that is we were unable to measure Ts (the temperature at which the entropy of the system reaches zero). Therefore, empirical equations to derive the forces contributing entropy cannot be applied in this case. Since entropy is the measure of the disorder of the system, a small change in entropy infers that no or small changes in conformation are observed in the experimental temperature range. In this context, major conformational changes are usually accompanied by large and positive entropic changes.

The change in heat capacity is an indication of structural changes as it is associated with changes in the surface area upon ligand binding. The change in heat capacity during the binding of GMP and UMP with the PAZ domain was very small and carried a positive sign, indicating that there were conformational changes accompanying substrate binding by the PAZ domain. Taken together, the small values of ΔS and ΔCp indicated that induced fit is the general mechanism for the binding of nucleotides with the PAZ domain. In order to validate this assumption, we calculated the average changes in the surface components of the PAZ domain using the available structures (Table S3). The observed changes in surface area between the Apo and cytidine nucleotide-bounded Drosophila Ago2 PAZ domain were very small (+350 Å). In addition, applying the polar and non-polar components to equation 2 yields a measured ΔCp of +6.4. Such a small calculated ΔCp value indicates the lack of significant conformational changes. A similar finding was observed after comparing the binding of the human EIF2C2/Ago2 PAZ domain with either U or G nucleotides, in which there was little or no change in the presence of various substrates. Such results correlate with the hypothesis that the RNA-binding cavity of the Ago1 PAZ domain is a preformed pocket 32. Thus, this feature seems to be conserved among Ago proteins.

On the basis of these observations, we assume that during RNAi, the PAZ domain moves as a single unit or whole molecule toward the 3′-end of the RNA with no or little internal conformational changes.

### Correlation between the measured binding affinity and the observed RNAi

The obtained RNAi data indicated the general preference of RNAi for ribonucleotides. A surprising issue in these data was the *in vivo* activity of siRNAs containing A nucleotides at their 3′-terminal, in spite of poor binding with the PAZ domain. In this context, siRNAs containing U or G were among the strongest inducers of RNAi at 1 and 10 nM, respectively. Within the known Ago proteins, the most catalytically active member is Ago2, while Ago1, Ago3, and Ago4 are inactive. Ago2 removes the passenger strand promptly; however, a major problem in RNAi experiments is that the other Ago proteins remain bound with the double-stranded siRNA for several days, thus, they slowly accumulate the guide strand [45]. Furthermore, little is known about the functional properties of other Ago proteins and this might complicate the *in vivo* results. Additionally, blunt-ended siRNA duplexes are also capable of inducing a gene silencing effect [46], [47]. When the role of the PAZ domain in RNAi was analyzed, it was reportedly essential for the specific and productive incorporation of siRNA into the RNAi pathway [21]–[23]. Importantly, PAZ-disrupted Ago mutants are unable to unwind or eject the passenger strand, thus highlighting the importance of the PAZ domain in the formation of a functional RISC complex [25]. Since ITC involves the direct measure of the association between two binding partners without any other interfering factor, we rely on ITC data to classifying the substrates according to their affinities. The effect of the strength of binding of siRNA with PAZ domain can be regarded as stage-specific matter. Stable complexes of 3′ terminal of siRNA with PAZ domain are required during binding of dsRNA, during unwinding and release of the passenger strand. This is also important for guiding the RISC complex to bind with the target mRNA. In contrast, the release of 3′ terminal of siRNA from PAZ domain is important for base pairing with target RNA and target cleavage.

In conclusion, the EIF2C2/Ago2 PAZ domain showed selectivity for ribonucleotides over deoxyribonucleotides. The highest binding affinity was shown by UMP, CMP, and GMP. There was little change in entropic conditions and heat capacity, indicating a more or less static preformed cavity for siRNA binding. The PAZ domain showed an inherent bias against adenine nucleotides, which showed low binding affinity. The strongest RNAi efficacy was shown for siRNAs containing uridine or guanosine nucleotides at their 3′-terminal. Finally, conservation of the 2′-OH or introducing a similar interaction seems to be essential for maintaining their high binding affinity with the PAZ domain.

## Supporting Information

Figure S1 Structure and NMR of 5-methyl uridine monophosphate.
(DOC)

Figure S2 Thermodynamic signatures of UMP, GMP, CMP, TMP and dGMP binding with Ago2PAZ domain.
(DOC)

Figure S3 the binding affinity of UMP, GMP, CMP, TMP and dGMP binding with Ago2PAZ domain.
(DOC)

Figure S4 Titration of Ago2PAZ domain with the rTMP. The top panel shows the raw calorimetric data referring to the amount of heat produced following each injection. The bottom panel shows the integrated amount of heat generated per injection as a function of the molar ratio of rTMP to protein.
(DOC)

Figure S5 Amino acid alignment from different Agos. The residues forming the cavity of nucleotides binding are included in green boxes. The residues of direct contact with nucleotides and enclosed by red boxes.
(DOC)

Table S1 The output of analyzed RNAi (1 nM) data by STATA. A single-tailed one-way analysis of variance with Bonferroni's multiple comparison test was conducted.
(DOC)

Table S2 The output of analyzed RNAi (10 nM) data by STATA. A single-tailed one-way analysis of variance with Bonferroni's multiple comparison test was conducted.
(DOC)

Table S3 The calculated accessible surface area parameters of Ago2PAZ domain. VADAR program is used in the calculations with a solvent probe radius of 1.4 Å.
(DOC)
