# Supplementary figures and images for: Bioenergetics and Gene Silencing Approaches for Unraveling Nucleotide Recognition by the Human EIF2C2/Ago2 PAZ Domain
Source: PLoS One. 2014 May 2;9(5):e94538. doi: 10.1371/journal.pone.0094538 (PMC4008379; doi:10.1371/journal.pone.0094538)

**Figure S1**

**
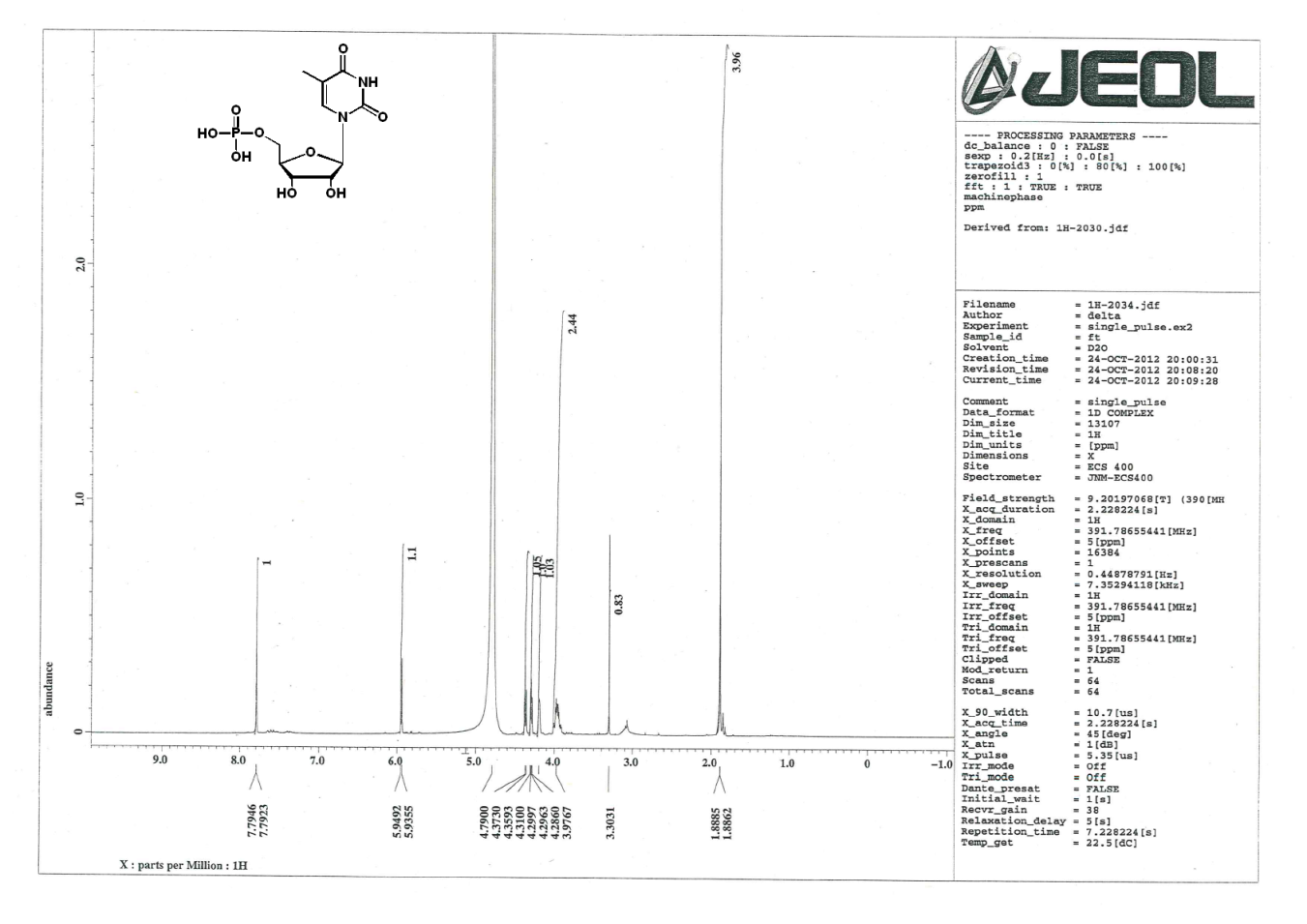
**

Supplement: Figure S1 — Structure and NMR of 5-methyl uridine monophosphate. (DOC) [file pone.0094538.s001.doc]

**Figure S2**


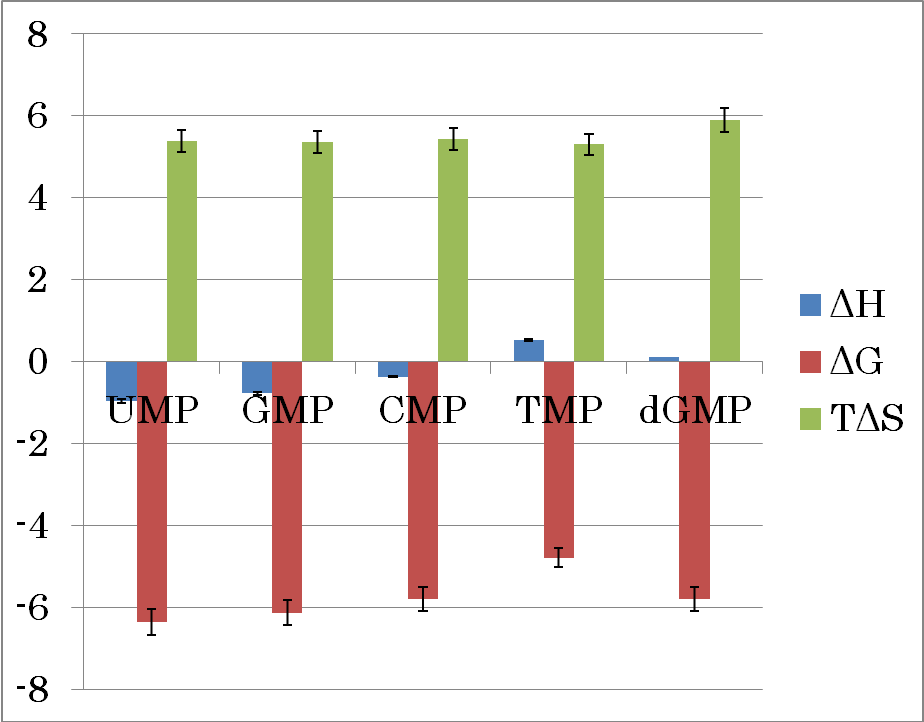

Supplement: Figure S2 — Thermodynamic signatures of UMP, GMP, CMP, TMP and dGMP binding with Ago2PAZ domain. (DOC) [file pone.0094538.s002.doc]

**Figure S3**


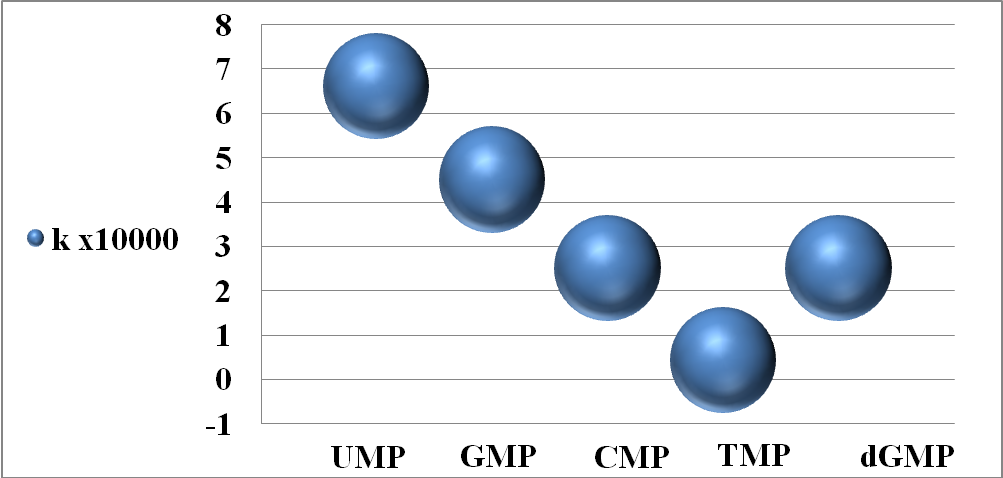

Supplement: Figure S3 — the binding affinity of UMP, GMP, CMP, TMP and dGMP binding with Ago2PAZ domain. (DOC) [file pone.0094538.s003.doc]

**Figure S4**

**
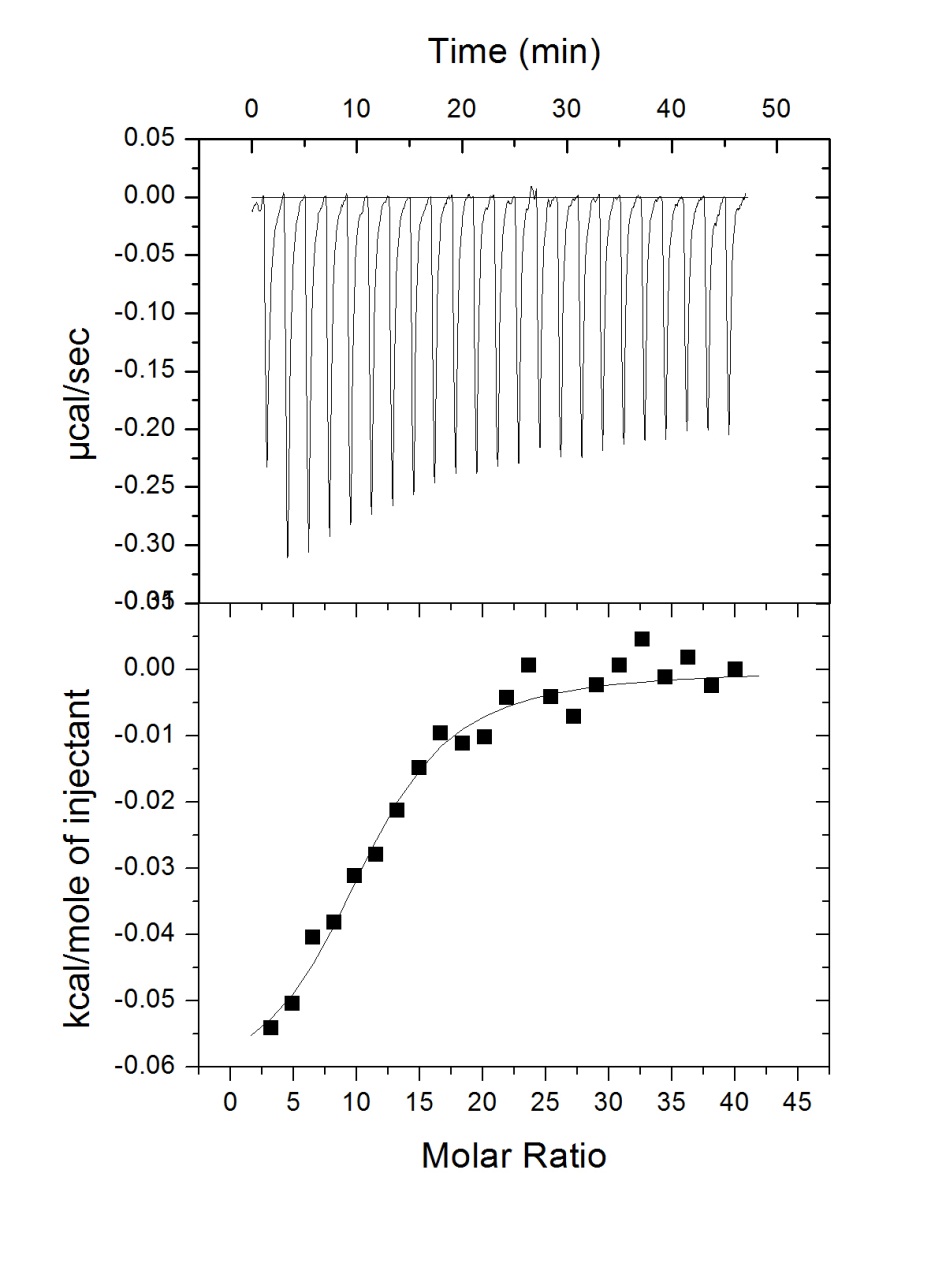
**

Supplement: Figure S4 — Titration of Ago2PAZ domain with the rTMP. The top panel shows the raw calorimetric data referring to the amount of heat produced following each injection. The bottom panel shows the integrated amount of heat generated per injection as a function of the molar ratio of rTMP to protein. (DOC) [file pone.0094538.s004.doc]

**Figure S5**


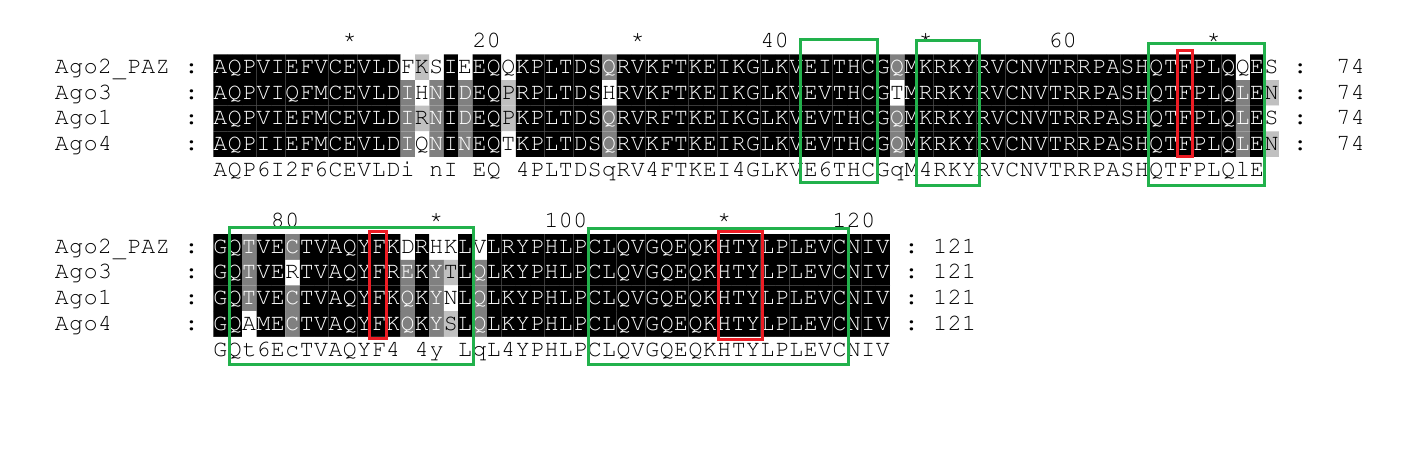

Supplement: Figure S5 — Amino acid alignment from different Agos. The residues forming the cavity of nucleotides binding are included in green boxes. The residues of direct contact with nucleotides and enclosed by red boxes. (DOC) [file pone.0094538.s005.doc]
