# Supplementary material for: Bioenergetics and Gene Silencing Approaches for Unraveling Nucleotide Recognition by the Human EIF2C2/Ago2 PAZ Domain
Source: PLoS One. 2014 May 2;9(5):e94538. doi: 10.1371/journal.pone.0094538 (PMC4008379; doi:10.1371/journal.pone.0094538)
Supplement: Table S1 — The output of analyzed RNAi (1 nM) data by STATA. A single-tailed one-way analysis of variance with Bonferroni's multiple comparison test was conducted. (DOC) [file pone.0094538.s006.doc]

**Table S1**

F< 0.0001

Prob > F 4.99

|  | control | U | dA | dC | dG | dT | rA | rC |
| --- | --- | --- | --- | --- | --- | --- | --- | --- |
| control |  | 0.319** |  |  |  |  |  |  |
| dA | -0.132 | 0.187 |  |  |  |  |  |  |
| dC | -0.047 | 0.272* | 0.084 |  |  |  |  |  |
| dG | -0.175 | 0.144 | -0.042 | -0.127 |  |  |  |  |
| dT | 0.076 | 0.243 | 0.056 | -0.028 | 0.098 |  |  |  |
| rA | -0.355** | -0.035 | -0.222 | -0.307* | -0.179 | -0.278* |  |  |
| rC | -0.31* | 0.009 | -0.177 | -0.262* | -0.134 | -0.233 | 0.044 |  |
| rG | -0.257 | 0.062 | -0.124 | -0.209 | 0.081 | -0.18 | 0.098 | 0.053 |

*Significant at 0.05 level

**Significant at 0.01 level
