# Supplementary material for: Bioenergetics and Gene Silencing Approaches for Unraveling Nucleotide Recognition by the Human EIF2C2/Ago2 PAZ Domain
Source: PLoS One. 2014 May 2;9(5):e94538. doi: 10.1371/journal.pone.0094538 (PMC4008379; doi:10.1371/journal.pone.0094538)
Supplement: Table S2 — The output of analyzed RNAi (10 nM) data by STATA. A single-tailed one-way analysis of variance with Bonferroni's multiple comparison test was conducted. (DOC) [file pone.0094538.s007.doc]

**Table S2**

F<0.0001

Prob > F 10.22

|  | control | U | dA | dC | dG | dT | rA | rC |
| --- | --- | --- | --- | --- | --- | --- | --- | --- |
| control |  | 0.391** |  |  |  |  |  |  |
| dA | -0.336** | 0.054 |  |  |  |  |  |  |
| dC | -0.287** | 0.103 | 0.048 |  |  |  |  |  |
| dG | -0.325** | 0.061 | 0.006 | -0.042 |  |  |  |  |
| dT | -0.358** | 0.032 | -0.022 | -0.07 | -0.285 |  |  |  |
| rA | -0.43** | -0.035 | -0.094 | -0.142 | -0.1 | -0.072 |  |  |
| rC | -0.351** | 0.001 | -0.055 | -0.103 | -0.061 | -0.033 | 0.038 |  |
| rG | -0.436** | -0.045 | -0.1 | -0.148 | -0.106 | -0.007 | -0.005 | -0.044 |

**Significant at 0.01 level
