# Supplementary material for: Bioenergetics and Gene Silencing Approaches for Unraveling Nucleotide Recognition by the Human EIF2C2/Ago2 PAZ Domain
Source: PLoS One. 2014 May 2;9(5):e94538. doi: 10.1371/journal.pone.0094538 (PMC4008379; doi:10.1371/journal.pone.0094538)
Supplement: Table S3 — The calculated accessible surface area parameters of Ago2PAZ domain. VADAR program is used in the calculations with a solvent probe radius of 1.4 Å. (DOC) [file pone.0094538.s008.doc]

**Table S3**

|  | Apo-PAZ (drosophila) | PAZ (Drosophila) bound with C at 3' terminal | PAZ (Human) bound with U at 3' terminal | PAZ (Human) bound with G at 3' terminal |
| --- | --- | --- | --- | --- |
| Pdb ID | 1vyn | 1t2r | 4ei1 | 3f3t |
| Total ASA | 7024.7 | 7375.6 | 8398.4 | 8870.6 |
| Fraction nonpolar ASA | 0.55 | 0.57 | 0.58 | 0.56 |
| Fraction polar ASA | 0.24 | 0.25 | 0.22 | 0.21 |
| Mean frac ASA | 0.3 | 0.28 | 0.3 | 0.3 |
